# Ethnicity and socioeconomic status: missing in research means missing in clinical guidance

**DOI:** 10.3399/BJGPO.2021.0034

**Published:** 2021-05-26

**Authors:** Eleanor Beard, Sharon Dixon, Tanvi Rai, Gail Hayward

**Affiliations:** 1 University of Oxford Medical School, Medical Sciences Division, University of Oxford, Oxford, UK; 2 Nuffield Department of Primary Care Health Sciences, University of Oxford, Oxford, UK

**Keywords:** Guidelines, Ethnic groups, Inequalities, Health Status Disparities, United Kingdom, COVID-19, General practice

## Introduction

As GPs, medical students and researchers serving diverse and disadvantaged communities, we are often frustrated by the lack of evidence for chronic disease management that is tailored to the populations we serve. The COVID-19 pandemic and the *Black Lives Matter* campaign have highlighted how racism and inequality persist in society and the impacts this has on health inequalities, including inequities in representation in clinical guidelines.

Type 2 diabetes is an excellent example of a condition which is socially patterned and is also more prevalent in Asian and Black patients compared to those of White ethnicity in the UK, with differences in morbidity and mortality.^1^ The metabolism, the therapeutic effectiveness of drugs, and the risk of adverse drug reactions can vary significantly between different ethnic groups.^2^ There is also ethnic variation in drug compliance and healthseeking behaviours, which can reflect structural inequities in healthcare delivery.^3^

The intersection of ethnic minority status and low socioeconomic status (SES) can compound the effects of the health inequalities linked to ethnicity. SES influences disease prevalence and patient response to treatment,^4^ with greater challenges meeting diabetic care targets in areas of deprivation.^5^

Clinical trials often fail to include representative proportions of certain groups, such as ethnic minorities, women, or those with a low SES.^6^ This means that clinical guidance is often based on evidence that has not explored the best treatment options for individuals from these underrepresented groups.^7^ For type 2 diabetes, this lack of representation of ethnic minority and low SES groups is particularly important as these groups are disproportionately affected by diabetes.

To understand how well ethnic minority groups and those from different SES bands are represented in the evidence informing clinical guidelines, we assessed the studies informing a review question from a NICE guideline regarding pharmacological therapies for type 2 diabetes.^8^ Considering its broad relevance, we identified Review question 1: ’*Which pharmacological blood glucose lowering therapies should be used to control blood glucose levels in people with type two diabetes?’*. In the 113 articles cited in the evidence tables, we assessed whether the ethnic and SES breakdown of the study sample had been recorded, and if the role played by ethnicity and SES was discussed in the study limitations.

### Reporting of ethnicity and SES breakdown of the study sample

**Figure 1.**
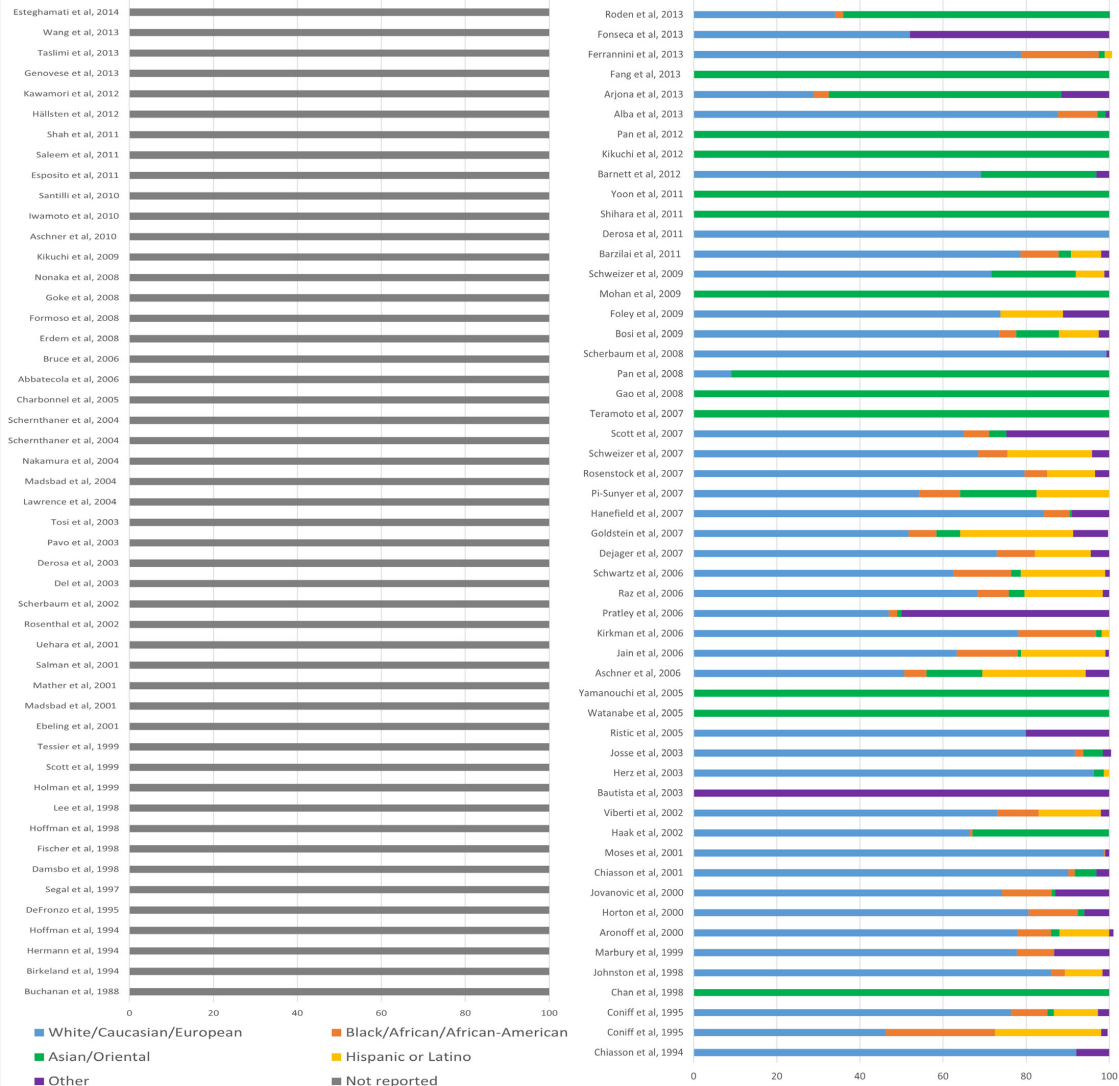
The breakdown of ethnic groups in the trials that informed the review questions of the NICE guideline. Ethnic categories are presented as documented in the original articles. The articles not reporting ethnicity are grouped on the left of the figure, and the other articles are listed on the right in descending order of the time of publication.

The guideline included 113 references. Despite help from a University of Oxford librarian and correspondence with NICE, we were unable to source 10 of these articles. Of the 103 articles assessed, 54 (52%) reported the breakdown of the study sample population based on ethnicity. Ethnicity was reported more frequently in more recently published articles; 54% of the articles published between 2000–2019 reported ethnicity, compared to 32% among those published between 1980–1999. Although 54 articles reported ethnicity among baseline characteristics, none of these articles reported trial outcomes in terms of these demographics. Figure 1 graphically depicts the breakdown of ethnic groups in the trials that informed the review questions of the NICE guideline, with the ethnicity labels as reported by the original articles. No studies explained how they determined ethnicity, and it is unclear whether these ethnic categories were self-identified, inferred by researchers or clinicians, or based on nationality.

No article reported socioeconomic status in the baseline characteristics, aside from a single article that reported education level (as a proxy) but did not report trial outcomes according to this variable.

### Representation of different ethnic groups

Of the studies reporting ethnicity, the percentage of different ethnic groups was largely matched in the control and intervention arms. Figure 1 shows the full ethnic breakdown of the trials assessed. It is difficult to directly compare individual studies due to the heterogenous ethnicity labels, but Figure 1 highlights the overall trends in the data supporting the NICE guideline. Thirty-six studies (67%) — based largely in European countries and the USA — were described as having a predominantly White, European, or Caucasian population, with a minority of other ethnicities. Fifteen studies based in East Asian countries reported a predominantly Chinese, Korean, or Japanese sample population. No study reported having a predominantly South Asian or Afro-Caribbean sample population.

### Implications for the future research and practice

Only eight articles acknowledged the limitations of under-representation of minority demographic groups. The NICE guideline did not acknowledge these limitations either. More recently published articles were more likely to report ethnicity. Collecting and reporting data on both ethnicity and SES should become part of the compulsory standard procedures in trial guidelines.^9^ However, ethnicity is not an internationally consistent or standardised measure, which itself can complicate the representation of ethnic groups in clinical trials. Nevertheless, developing a high quality, transparent and internationally replicable approach to ascertaining and reporting ethnicity and SES (and showing how it is interpreted for that research question) should be a priority for future research and policy.

No article provided data on study attrition, and it would be useful to determine whether there are barriers to both accessing trials initially and sustaining longer-term involvement based on ethnicity and/or SES.

Clinical guidelines are designed to aid decision making, improve patient outcomes, and ensure a similar standard of treatment provision throughout healthcare institutions.^10^ Clinicians are less likely to follow guidance if they believe they are poorly developed or not relevant to their patients.^10^ This can lead to a lack of continuity in the delivery of health care, and may contribute to unequal health outcomes. The accurate representation of the clinical population in clinical studies will support the development of better guidelines, thus enabling clinicians to make evidence-based decisions for their patients. We recognise that the choice of a specific drug regime to personalise glucose control is influenced by a range of factors, such as weight, sex, and renal function.^11^ Reporting of ethnicity and SES, and determining their relationship with treatment outcomes, may not be sufficient to personalise treatment outcomes, but it will be a positive development in optimising guidance for a wider range of demographic groups. The movement towards a more explicit assessment of trial samples is already underway, as evident from the NIHR INCLUDE guidance for representation of under-served groups.^9^

## Conclusion

Reporting of ethnicity is lacking in almost half of the studies contributing to a NICE guideline question about pharmacological management of type 2 diabetes (and SES reporting is almost completely absent).

Where reported, sample populations often failed to represent the true clinical population demographics, meaning that there is limited evidence to support current guidelines for the true clinical population. We have assessed only one domain of a NICE guideline for one disease, but the principles are likely to apply to the evidence base informing many guidelines. We call for standardised approaches to report ethnicity and SES, as well as guidelines for the representation of different ethnic groups and SES bands in all study populations. Taking steps to address the current deficit of evidence for the clinical outcomes in these underrepresented groups will offer clinicians better tools to optimise care and could help to reduce health inequalities.
